# Narcolepsy and risk of cardiovascular outcomes: getting to the heart of the matter

**DOI:** 10.1093/sleep/zsaf244

**Published:** 2025-08-18

**Authors:** Alessandro Silvani

**Affiliations:** Department of Biomedical and Neuromotor Sciences, University of Bologna, Bologna, Italy

Globally, cardiovascular diseases (CVD) are the leading cause of death, accounting for at least 38% of premature deaths under the age of 70 attributable to noncommunicable diseases [1]. The prevention of CVD is a public health priority. The American Heart Association (AHA) has recognized healthy sleep as a key component of cardiovascular health as part of its “Life’s Essential 8” framework [2]. The AHA further underscored the connection between sleep disorders and cardiovascular health by publishing a Fact Sheet that specifically highlights the impact of obstructive sleep apnea (OSA), insomnia, restless legs syndrome (RLS), and narcolepsy [3]. The inclusion of narcolepsy, a rare disease [4], among these more prevalent sleep–wake disorders reflects a growing body of evidence linking it to several cardiovascular risk factors and comorbidities [5].

In a recent study, Riaz et al. contributed significantly to this evidence base [6]. They conducted a large-scale analysis of the MarketScan Commercial Claims and Encounters and the Medicare Supplemental Databases, spanning a 17-year period from 2005 to 2021. Their study involved a cohort of 34 562 patients with a new diagnosis of narcolepsy who were propensity-score matched with 100 405 controls without narcolepsy or hypersomnia. The matching process accounted for baseline demographics and comorbidities, including diabetes, hypertension, hyperlipidemia, and obesity, for co-occurring sleep–wake disorders, including insomnia, OSA, and RLS, and for use of medications. Critically, Riaz et al. controlled for the use of wake-promoting agents, psychostimulants, and oxybate as covariates in their multivariate models [6], which is relevant because these medications may increase blood pressure in patients with narcolepsy [7, 8]. After adjusting for these covariates, patients with narcolepsy had a significantly increased risk of developing CVD (including stroke, atrial fibrillation, heart failure, myocardial infarction, and acute coronary syndrome), with an adjusted hazard ratio of 1.89 (95% CI = 1.71 to 2.09) compared to matched controls. A similar result was obtained for major adverse cardiovascular events [6].

The findings from Riaz et al. [6] are consistent with prior research by Ben-Joseph et al., who, using a different statistical approach on the same MarketScan database, reported similar associations, albeit with a slightly lower adjusted hazard ratio for CVD (1.30, 95% CI = 1.08 to 1.56). Notably, their study did not adjust for the use of wake-promoting agents, psychostimulants, or oxybate [9]. The findings from Riaz et al. [6] also align with those from a separate report from the same group on the MarketScan database. In that study, Kaufmann et al. focused on the time to the first occurrence of hypertension, hyperlipidemia, diabetes, and nonalcoholic fatty liver disease/ nonalcoholic steatohepatitis as primary outcomes [10]. The results showed that patients with narcolepsy, including those in the youngest age group (<25 years), had an increased risk for developing hypertension, hyperlipidemia, and diabetes compared to matched controls [10].

Narcolepsy is a heterogeneous condition comprising narcolepsy type 1 (NT1) and type 2 (NT2). Both are defined by excessive daytime sleepiness (EDS) [11], which leads to the hypothesis that EDS may underlie the increased CVD risk in patients with narcolepsy. Accordingly, individuals with OSA and objective EDS at baseline, as measured by the multiple sleep latency test, have a higher mortality rate from CVD compared to those without either condition (adjusted hazard ratio: 3.88; 95% CI = 1.29 to 11.62) [12]. However, multiple pathophysiological pathways may contribute to EDS, and the extent of their overlap between OSA and narcolepsy is unclear [13].

NT1 is distinguished from NT2 by the typical presence of cataplexy and by a profound deficiency of the orexin neuropeptides, resulting from the near-complete functional loss of orexin-producing neurons [11]. Polysomnography reveals that NT1 is also associated with shallow and fragmented nocturnal sleep, in contrast to idiopathic hypersomnia. The sleep macrostructure of NT2 and idiopathic hypersomnia without long sleep time, however, is remarkably similar [14]. This fits with the proposal that these two conditions should be reclassified as a single entity, termed “narcolepsy spectrum disorder,” which could represent a continuum between NT1 and idiopathic hypersomnia [15].

A crucial area of investigation is to differentiate the CVD risk profiles of NT1 and NT2, which could elucidate the roles of severe orexin deficiency and sleep fragmentation—both of which are supported by robust basic and translational research. In murine models of NT1, orexin deficiency leads to persistent sympathetic activation during sleep with respect to wild-type mice, resulting in a less effective decrease in blood pressure during sleep with respect to wakefulness [16,17]. In patients with NT1, this manifests as a non-dipping blood pressure profile [18,19], which is an independent risk factor for CVD in the general population [20]. Furthermore, orexin deficiency in mice increases circulating Ly-6C^high^ monocytes during the rest period, thereby significantly accelerating atherosclerosis in genetically and environmentally predisposed animals [21]. This effect is not exclusive of complete orexin deficiency, as partial reduction of brain orexin levels induced by chronic experimental sleep fragmentation also increases atherosclerosis [21]. Chronic sleep fragmentation also causes hypertension, arterial stiffness, endothelial impairment, and vascular remodeling in mice [22].

Contrary to what might be expected, the sensitivity analyses conducted by Riaz et al. revealed a remarkably similar increase in CVD risk for both NT1 and NT2 [6]. These findings are consistent with the results from the studies by Ben-Joseph et al. [9] and by Kaufmann et al. [10]. However, these conclusions should be interpreted with caution. The smaller sample size of patients with NT1 compared to NT2 may have limited the statistical power to detect significant differences, and a major limitation of studies using insurance claims databases is the lack of specific laboratory values needed to confirm diagnoses or differentiate between narcolepsy types. This data limitation may contribute to explain why the NT2/NT1 sample size ratio in the study from Riaz et al. [6] was much higher (5.2 vs. 0.2) than the ratio reported in 2018 in the European Narcolepsy Network database [23] and in a study on narcolepsy prevalence in Catalunya, Spain (7+ million inhabitants) [24], both of which included patients carefully evaluated by narcolepsy experts. The NT2/NT1 ratio in the study from Riaz et al. [6] was also higher than the corresponding ratios of 3.3 and 2.0 reported by Ben-Joseph et al. [9] and Kaufmann et al. [10], respectively. Furthermore, while the pathophysiology of NT2 is not fully understood, some evidence suggests it may involve a partial and localized loss of orexin neurons [25]. As previously noted, even a partial orexin deficiency is sufficient to promote atherosclerosis in mice [21], which could account, at least in part, for a similar CVD risk profile between NT1 and NT2.

In conclusion, the findings from Riaz et al. [6] strengthen the rationale for prioritizing CVD prevention in patients with narcolepsy [5, 26]. However, this study does not definitively identify the mechanisms responsible for the increased CVD risk in patients with narcolepsy. The persistence of this risk even after statistically controlling for traditional cardiovascular risk factors, sleep comorbidities, and use of medications suggests a more complex pathophysiology. To elucidate this pathophysiology, and particularly the distinct causal contributions of EDS, orexin deficiency, and sleep fragmentation, future research will need to overcome the significant challenge of acquiring detailed clinical and laboratory data on large cohorts of patients despite the rarity of narcolepsy. This may require expanding large-scale collaborations among clinical centers and leveraging basic research on animal models to demonstrate causal links. Understanding these root causes is crucial not only for narcolepsy, especially in light of promising clinical trials for orexin receptor agonists [27], but also for other sleep–wake disorders and for public health more broadly.
